# Screening of specific diagnostic peptides of swine hepatitis E virus

**DOI:** 10.1186/1743-422X-6-186

**Published:** 2009-11-04

**Authors:** Kai Zhao, Qiwen Liu, Ruisong Yu, Zhen Li, Jianyue Li, Hong Zhu, Xiao Wu, Furong Tan, Jinbin Wang, Xueming Tang

**Affiliations:** 1 Biotechnology Research Institute, Shanghai Academy of Agricultural Sciences, 2901 Beidi Road, Shanghai, 201106, PR China; 2 Key Laboratory of Agricultural Genetics and Breeding, Shanghai Academy of Agricultural Sciences, 2901 Beidi Road, Shanghai, 201106, PR China; 3 College of Life and Environment Sciences, Shanghai Normal University,100 Guilin Road, Shanghai 200234, PR China; 4 Institute of Animal Science and Veterinary Medicine, Shanghai Academy of Agricultural Sciences, 2901 Beidi Road, Shanghai, 201106, PR China

## Abstract

**Background:**

Swine hepatitis E virus (swHEV) is a zoonotic disease that is considered a major problem in pig production and presents a threat to human health. Elucidation of the major antigenic epitopes of swHEV is essential for the effective control of swHEV epidemics.

**Results:**

By bioinformatic analysis, we identified and then synthesized 12 peptides from open reading frames (ORFs) ORF1, ORF2 and ORF3, including swHEV-1 - swHEV-12. Using the results from ELISA, we selected swHEV-11 as the best candidate antigen and used it as a coating antigen for the development of peptide-based swine anti-HEV ELISA kits. The coefficient of variation (CV) the coefficient of variation (CV) varied between 4.3-7.2% in the same batch, and between 8.2-17.7% in six different batches. When comparing our swine peptide-based kit with the commercial recombinant-based kit, the humane anti-HEV IgG test had a 73.4% correspondence rate for them.

**Conclusion:**

This is the first systemic study to screen the diagnostic peptides of swHEV and our findings strongly suggest that peptide swHEV-11 is a potent diagnostic reagent of swHEV that could be used in the development of highly efficient diagnostic assays for the specific and highly sensitive detection of anti-HEV activity in swine serum samples.

## Background

Swine hepatitis E virus (swHEV) was discovered in pigs in the USA in 1997 [1], and it has since been demonstrated that there is potential for the zoonotic transmission and cross-species transmission between humans and pigs [2]. Based on phylogenetic analysis, swHEV genotypes 3 and 4 contain genomic sequences closely related to human HEV [3,4]. To date, swHEV infection has been documented in a number of provinces and municipalities in China [5], and is considered a major problem in pig production and a threat to human health. There is therefore a real need for specific and effective methods of diagnosis, prophylaxis and treatment for this disease.

The antigenic structure of the related human HEV virus has been studied in detail. Several antigenic regions of diagnostic relevance were found within the open reading frames (ORFs) ORF1, ORF2 and ORF3, using a range of different sized synthetic peptides [6-10] or recombinant proteins [11-13]. Three of the seven peptides encoded by ORF1, ORF2 and ORF3 of human HEV were found to be immunogenic [14]. pB166, comprising overlapping recombined peptides from ORF2, is a neutralization epitope that has the potential to be used in the development of vaccines to prevent human HEV [15]. The peptide comprising 11 amino acids from the C-terminus of human HEV-virus-like particles (HEV-VLP) is immunogenic [16], and can be used to elicit antibodies.

Some preliminary studies have investigated the antigenic epitopes of swHEV. Solid-phase peptide synthesis was used to generate peptides from swHEV ORF2, and this report provided a useful method for the detection of swHEV in formalin-fixed, paraffin-embedded tissues [17]. However, the antigenic structure of swHEV is yet to be fully investigated. The research on swHEV has been limited mainly to local epidemiological studies and simple clinical analysis of animals. The commercially available kits to detect swHEV are the same as the kits used to detect human HEV. Despite the fact that there is high homology between the genomic sequences of swHEV and human HEV, there are still important differences and minor changes could affect the sensitivity and specificity of the diagnostic test when applied to animals. Therefore, the results obtained from using a human HEV epitope to detect swHEV are not very satisfactory.

In this study, we analyzed antigens of HEV using the DNA Star software. Twelve peptides were found to be synthesized from the three ORFs. Based on the results of ELISA tests, we selected peptides that displayed antigenicity, of which peptide swHEV-11 was the best candidate. We then used this antigenic peptide as a coating antigen to develop an ELISA kit for the rapid diagnosis of swHEV. The results from this study suggest that peptide swHEV-11 is an effective diagnostic reagent that can be used in the development of more efficient diagnostic assays for the detection of anti-HEV activity in swine serum samples.

## Results

### Examination of the Swine sera specimens

The sera collected from a Shanghai abattoir were confirmed using a commercial human anti-HEV kit (Beijing WANTAI Biological Pharmacy Enterprise Co. Ltd.). We detected sera that were positive for swHEV IgG and IgM antibodies and sera that were negative for swHEV IgG and IgM antibodies. In this experiment, we identified six negative sera and 110 positive sera.

### Hydrophobicity and secondary structure of HEV proteins and identification of synthetic peptides

**Figure 1:**
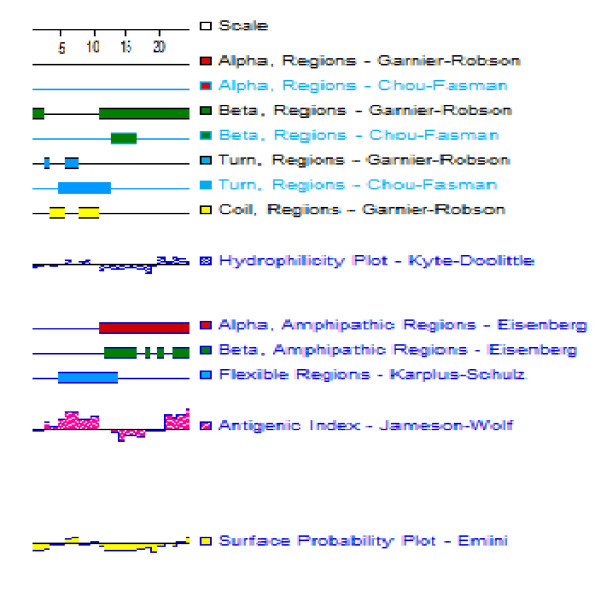
**Hydrophobic analysis and secondary structure prediction of encoding proteins of swHEV11 of ORF3**.

The analysis of hydrophobicity and the prediction of secondary structure for HEV non-structural (ORF1) and structural (ORF2 and ORF3) proteins predicted that certain regions have stronger antigenicity (Figure 1), even though the potential antigenic epitopes were widely distributed along the entire amino acid sequence. Twelve areas were chosen across the 3 ORFs and peptide sequences were designed based on the parameters of hydrophobicity, B-turn and B-sheet secondary structures. These 12 fragments of swHEV peptides were then synthesized.

**Figure 2:**
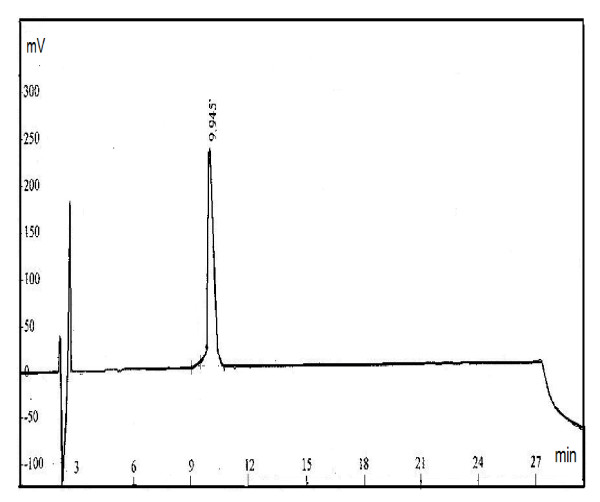
**Purity profile of synthetic HEV peptide-swHEV11 on HPLC**. Column, Kromasil-C_18_(4.6 × 250 nm); wavelength, 220 nm; buffer A, 0.1% TFA in 100% water; buffer B, 0.1% TFA in 100% acetonitrile; flow rate, 1.0 ml/min; gradient, from 0 to 75% of solution A.

The synthesized HEV peptides were purified further by preparative HPLC. Figure 2 shows the purity profile of synthetic HEV peptide, swHEV-11, which had a value of 96.7%. The amino acid composition analyses of these peptides proved to be consistent with their expected values.

### Selection and Confirmation of peptides

The immunoreactivity of the synthetic peptides with swHEV serum specimens demonstrated that eight of the peptides: swHEV-2 and swHEV-3 (from ORFl), swHEV-5 and swHEV-7 (from ORF2), swHEV-9, swHEV-10, swHEV-11 and swHEV-12 (from ORF3), had antigenicity against IgG antibody. These eight peptides detected 17, 19, 11, 14, 18, 13, 21 and 21 positive references, respectively, out of 24 positive sera (Table 1). The positive rates of these references were 70.8, 79.2, 45.8, 58.3, 75.0, 54.2, 87.5 and 87.5%, respectively. The eight peptides also detected 3, 4, 5, 4, 3, 5, 6 and 4 negative references, respectively, out of six negative sera, and the rates detected were 50, 66.7, 83.3, 66.7, 50, 83.3, 100 and 66.7%, respectively (Table 1). When IgM antibody was used, the peptides detected 4, 5, 3, 0, 6, 6, 5, 5 and 3 positive references, respectively, out of 24 positive references (Table 2).

**Table 1:** Immunoreactivity of synthetic swine HEV peptides (for IgG) with a positive serum panel and a negative serum panel

Reference	Positive references		Quantity	Negative references		Quantity
sera	+	-		+	-	
swHEV-1	6	18	24	0	6	6
swHEV-2	17	7	24	3	3	6
swHEV-3	19	5	24	2	4	6
swHEV-4	4	20	24	0	6	6
swHEV-5	11	13	24	1	5	6
swHEV-6	6	18	24	0	6	6
swHEV-7	14	10	24	2	4	6
swHEV-8	8	16	24	0	6	6
swHEV-9	18	6	24	3	3	6
swHEV-10	13	11	24	1	5	6
swHEV-11	21	3	24	0	6	6
swHEV-12	21	3	24	2	4	6

+, positive; -, negative

**Table 2:** Immunoreactivity of synthetic swine HEV peptides (for IgM) with a positive serum panel

Reference	Positive references		Quantity
sera	+	-	
swHEV-1	4	20	24
swHEV-2	5	19	24
swHEV-3	3	21	24
swHEV-4	0	24	24
swHEV-5	6	18	24
swHEV-6	6	18	24
swHEV-7	5	19	24
swHEV-8	5	19	24
swHEV-9	3	21	24

+, positive; -, negative

From all the above data, we confirmed swHEV-11 as the most antigenic of the 12 peptides and used this peptide in further studies.

### Reproducibility of the synthetic peptide-based test kits

Swine serum samples with anti-HEV IgG and IgM reactivity, were used for checking the reproducibility of this peptide-based swine anti-HEV ELISA kits. On analysis of reproducibility of the anti-HEV IgG test kit, the coefficient of variation (CV) varied between 4.3-7.2% in the same batch, and between 8.2-17.7% in six different batches (Table 3).

**Table 3:** Reproducibility of the peptide-based ELISA kits(Anti-HEV IgG kit)

Serum	Intro-batch^a^			Inter-batch^b^		
	X	**SD**.	CV	X	**S.D**.	CV
1	0.382	0.0209	5.5%	0.387	0.0475	12.3%
2	0.299	0.0215	7.2%	0.327	0.0468	14.3%
3	0.227	0.0134	5.9%	0.274	0.0223	8.2%
4	0.356	0.0152	4.3%	0.382	0.0423	11.1%
5	0.329	0.0185	5.6%	0.395	0.0450	10.4%
6	0.333	0.0163	4.9%	0.381	0.0670	17.7%

X, mean OD values; SD., standard deviation;

CV, coefficient of variation.

^a ^The intro-batch duplicateivity test

^b ^The inter-batch duplicativity test

### Comparison of synthetic peptide-based kit torecombinant-based kit

In a parallel experiment, 94 swine serum samples were used to compare the the peptide-based swine anti-HEV ELISA kits with the recombinant-based kit. The results indicated that 69 (including 64 positive and five negative) of the 94 samples (73.4%) were detected identically by anti-HEV IgG antibodies by both kits (Table 4), whereas 34 (including 29 positive and five negative) of the 94 samples (36.2%) were detected identically by anti-HEV IgM antibodies by both kits (Table 5).

**Table 4:** Comparison between the synthetic peptide-based and recombinant-based kits a for anti-HEV IgG test

Recombinant-based kit^a^	Peptide-based kit		
Subtotal			
	+	-	
+	64	24	88
-	1	5	6

Total			94

+, positive; -, negative.

^a ^From Beijing WANTAI Biological Pharmacy

Enterprise Co., Ltd.

**Table 5:** Comparison between the synthetic peptide-based and recombinant-based kits a for anti-HEV IgM test

Recombinant-based kit^a^ Subtotal	Peptide-based kit		
	+	-	
+	29	59	88
-	1	5	6

Total			94

+, positive; -, negative.

^a ^From Beijing WANTAI Biological Pharmacy

Enterprise Co., Ltd.

However, when compared the KHB kit (HRP-goat anti-pig IgG for 1:6000) with the recombinant-based kit from WANTAI, Beijing, the results indicated that 55 (including 50 positive and 5 negative) of 94 samples were identical in terms of both kits by the detection of anti-HEV IgG, with 58.5% of correspondence rate for anti-HEV IgG test (Table 6). We also compared the peptide-based kit with the KHB kit (HRP-goat anti-pig IgG for 1:6000). The results indicated that 55 (including 40 positive and 5 negative) of 94 samples were identical in terms of both kits by the detection of anti-HEV IgG, with 47.9% of correspondence rate for anti-HEV IgG test (Table 7).

**Table 6:** Comparison between the KHB kit and recombinant-based kits^a^

Recombinant-based kit a	KHB	kit b	Subtotal
	+	-	
+	50	38	88
-		5	6

Total	1		94

+, positive; -, negative.

^a ^From Beijing WANTAI Biological Pharmacy

Enterprise Co., Ltd.

^b ^From Shanghai Kehua Bio-Engineering Co., Ltd.

**Table 7:** Comparison between the synthetic peptide-based and KHB kit for anti-HEV IgG test

*KHB kit*^b^	*Peptide-based kit*		*Subtotal*
	+	-	
+	40	48	88
-	1	5	6

Total			94

+, positive; -, negative.

^b ^From Shanghai Kehua Bio-Engineering

Co., Ltd.

### Application of the peptide-based kit in the detection of HEV infection

The peptide-based swine anti-HEV ELISA kit was used to test 1564 swine specimens. 1142 of the 1564 swine serum samples (72.9%) were found to be positive for anti-HEV IgG antibodies (data not shown). The results demonstrated that the peptide-based anti-HEV ELISA kit is of high specificity in the detection of serum anti-HEV.

## Discussion

Detection of anti-HEV antibodies have shown that several animal species, including pigs and rodents, have high infection rates of HEV [18,19]. The seropositive rate was 82.5% in sows, 53.9% in 4- to 6-month-old swine, 63.4% in 1- to 3-month-old swine, with an overall rate of 55.7% for swine in an abattoir in eastern China [20].

The availability of swHEV detection assays that are sensitive and specific are essential for the swine industry and for research purposes. Serological diagnosis of HEV is more specific and robust than diagnosis via RNA detection [21]. The main problem with PCR detection of HEV is that, usually, viremia shedding in faeces is short-lasting and therefore the opportunity to detect the virus is limited. In animals, other samples where the virus could be detected more easily, such as the liver or bile, are most often only available in post-mortem. For this reason, serological tests such as ELISA are widely used [19].

The major antigenic epitopes of HEV located in ORF2 and ORF3 [8,11,22], should be the ideal diagnostic reagents of HEV detection [23]. A previous study used gene-I full-length HEV ORF3 antigenic protein in ELISA and detect HEV reference serum, the results of IgG antibody tests showed levels of sensitivity and specificity consistent with the national requirements [24]. These results were better than those obtained with Genelab reagents to detect clinical serum.

With the cloning of the Burmese and Mexican isolates of HEV [25-27], it is now possible to analyze the hydrophobicity and to predict the secondary structures of putative human HEV proteins [6,8,11]. On that basis, we selectively synthesized a set of 12 peptides from swHEV according to the sequence of strain swCh25 [28], a Chinese Xinjiang isolate, and used them to develop the peptide-based ELISA kits for the detection of swine anti-HEV.

The findings that the diagnostic test using IgG anti-HEV was more effective at screening for acute HEV than the diagnostic test using IgM anti-HEV [29], and that the short duration of anti-HEV IgM antibodies in serum occurs only from late incubation periods to early acute phases of the disease, resulted in the test system for the detection of anti-HEV IgG being regarded as the most effective approach for immunodiagnosis of HEV infection [14].

Synthetic viral peptides have been used in structure-function studies on viral genomes and their encoded proteins because the length, sequence and conformation of the peptides can be modified according to requirements [30].

Tests on the reproducibility of the synthetic peptide-based test kits suggest that the anti-HEV ELISA kits appear to be both stable and reliable. A series of experiments were carried out to assess the accuracy of the kits and to compare them with the recombinant-based kits, after first determining the cut-off values for the evaluation of the test results. The commercial kit detected human IgG, IgM and IgA, while our peptide-based kit detected IgG only, so the rate of detection of positive serum by our kit was lower than that of the commercial kit. Some specimens were reactive with more recombinant antigens than synthetic peptides, which may have been due to false positives obtained with ELISA using recombinant antigens or due to false negatives obtained with synthetic peptide ELISA resulting from conformational differences [23]. The KHB kit is used to detect human HEV, so the results are not very consistent with peptide-based kit in this experiment. The commercial kit was inefficient for the serodiagnosis of animals due to poor sensitivity [19]. In comparison, results with our peptide-based ELISA kit suggest that it is as good as the recombinant-based kit, and can be used for serodiagnosis of swHEV infection. Our peptide-based kit is sensitive and attains the requirements for clinical diagnostic use.

## Conclusion

The results obtained in this study strongly suggest that the peptide swHEV-11 from ORF3 is a potent diagnostic reagent of swHEV and can be used as a diagnostic target for the development of highly efficient prototype diagnostic assays for the specific and sensitive detection of anti-HEV activity in swine serum samples. The study of the swHEV-11 epitope might not only provide an efficient way to develop a kit to detect swHEV rapidly and accurately, but might also be applied to the development of vaccines for swHEV to control the spread of HEV and the prevention of this virus passing from pigs to humans. To our knowledge, this is the first systemic study to screen the diagnostic peptides of swHEV and is also the first demonstration that swHEV-11 from ORF3 could be used as a specific and highly sensitive diagnostic reagent.

## Materials and methods

### Synthesized Peptides and reagents

Peptides consisting of the antigens swHEV-1-4 from ORF1, swHEV-5-7 from ORF2 and swHEV-8-12 from ORF3, were synthesized by GL-Biochem Ltd. (Shanghai, China), the WANTAI kit (Beijing WANTAI Biological Pharmacy Enterprise Co. Ltd., China) (Table 8) and strain swCh25 sequence [GenBank: AY594199] of swine HEV.

**Table 8:** Amino acid sequence of each of the 12 peptides swHEV-_1,2,3,4,5,6,7,8,9,10,11,12_

Peptides	Sequence	Position
ORF1		
swHEV1	AGRCLEVGAHPRSINDNPNVLHRCFLKPVG RDVQRWYTAPTRGPAANCRRSALRGLPP	83-140
swHEV2	VEHNPKRLEAAYRETCSRRGTAAYPLLGA GIYKVPVGLSFDAWERNHRPGDE	874-925
swHEV3	SDSVLTFELTDIVHCRMAAPSQRKAVLSTL VGRYGRRTKLYEAAHADVRGS	1295-1346
swHEV4	ESLRGFWKKHSGEPGTLLWNTVWNMAVIAHC YEFRDLKVAAFKGDDSVVLCSDYRQSRDAAA	1521-1582
ORF2		
swHEV5	PRQPARPLGSAWRDQSQRPAASTRRRPAPAGA SPLTAVAPAPDTAPVPDVDSRGAILRRQYNLS	90-153
swHEV6	AQQDKGIAIPHDIDLGESRVVIQDYDNQ HEQDRPTPSPAPSRPF	433-476
swHEV7	PVSISAVGVLAPHSALAILEDTADYPAR AHTFDDFCPECRSLGLQGC	606-653
ORF3		
swHEV8	CPRHRPVSPLAVAAGGAAAVPAVVSGV TGLILSPSPS	22-58
swHEV9	SPSPSPIFIQPTPSHLTFQPQPGLELALGSQ PVHSAPLGATSPSAPPLPPVVDLPQLG	55-112
swHEV10	LALGSQPVHSAPLGATSPSAPPLPPVVD LPQLGLRR	79-114
swHEV11	LGATSPSAPPLPPVVDLPQLGLRR	91-114
swHEV12	LALGSQPVHSAPLGATSPSAPPL	79-101

### Swine sera specimens

Swine blood was collected from an abattoir in Shanghai (China) and centrifuged to isolate the sera. The sera were then confirmed using a commercial anti-HEV kit (Beijing WANTAI Biological Pharmacy Enterprise Co. Ltd.). We detected positive sera which were also positive for swHEV IgG and IgM antibodies, and negative sera which were also negative for swHEV IgG and IgM antibodies. Sera were stored at 4°C for later use.

### Computer analysis and chemical synthesis of HEV-encodingproteins

The antigenic structures relating to the sequences of HEV peptides from each ORF were analyzed using DNAStar software (Madison, Wisconsin, USA) and the hydrophilicity, surface tenacity, helix of the Chou-Fasman, lamellar and reverse turn, secondary structure of the Robson-Garnier and C- and N- termini of the protein and polypeptide amino acid sequences, and the Antigenic Index (AI) were predicted. We used DNAStar software and the amino acid sequence of each ORF to determine the AI (which was 10-15 amino acids) and assistant in the identification of antigen sequences.

### Coating and blocking of synthetic peptides

Synthetic peptides were diluted with a coating buffer (0.05 mol/l carbonate solution, pH 9.6), to a final concentration of 3 μg/ml. We added 100 μl of coating buffer to each microwell of a microtiter plate and plates were incubated at 4°C for at least 12 h. The microtiter plates were then blocked with 200 μ1 of tris-buffered saline (TBS) containing 3% bovine serum albumin (BSA) and incubated at 37°C for 2 h. Plates were then rinsed and dried for later use.

### Anti-HEV ELISA and calculation of cut-off values

In each peptide-coated microwell, 100 μl of TBST containing 1% BSA and 10 μl (or 1 μl for IgM) of swine sera specimen, were mixed and incubated at 37°C for 1 h. The microwells were washed five times with TBST. Then 100 μl of horseradish peroxidase-conjugated goat anti-pig IgG_h+l _(or IgM) specific for the γ-chain (or IgM specific for the μ-chain) at a 1:10,000 dilution (in TBST containing 1% BSA) were added as detection antibodies (Immunology Consultants Laboratory, Inc., Newberg, USA.) and incubated for 1 h at 37°C. After washing the microwells five times, color was developed by the addition of 100 μl per well of a substrate solution containing 3,3',5,5'-tetramethylbenzidine (TMB) and H_2_O_2_. After a 10-min incubation in the dark at 37°C, the reaction was stopped by the addition of 50 μl of 1 N HCl to each well. The optical densities (ODs) were measured at 450 nm.

In this experiment, we used negative sera, which had been detected using the WANTAI kit, as a negative control. Typically, the cutoff value, expressed as the P/N ratio, where P represents the optical density value at 450 nm of the experimental serum and N represents the optical density value at 450 nm of the negative controls, was established using ELISA analysis software Ezy-immuno and was equal to 2.1. If the rate was above 2.1, the experimental sera were classed as positive, whereas if the rate was lower than 2.1, the experimental sera were classed as negative.

## Abbreviations

HEV: hepatitis E virus; swHEV: swine hepatitis E virus; ELISA: enzyme-linked immunosorbent assay; ORF: open read fram; WANTAI: Beijing WANTAI Biological Pharmacy Enterprise Co., Ltd.; TBST: Tris-Buffered Saline Tween-20.

## Competing interests

The authors declare that they have no competing interests.

## Authors' contributions

KZ and QL participated in the design and carried out the majority of the experiments in the study and drafted the manuscript. SY, JL, HZ, XW, FT, JW and XT helped to carry out the experiments and draft the manuscript. ZL conceived of the study, participated in its design and coordination. All authors read and approved the final manuscript.
